# Reality shock in radiography: fact or fiction? Findings from a phenomenological study in Durban, South Africa

**DOI:** 10.1186/s40359-019-0317-9

**Published:** 2019-06-25

**Authors:** Tawanda Gilbert Alfred Chipere, Pauline Busisiwe Nkosi

**Affiliations:** 1 Department of Radiography, Faculty of Health Sciences, Durban University of Technology, P.O. Box 1334, Durban, South Africa; 2 Department of Radiography, Faculty of Health Sciences, Durban University of Technology, P.O. Box 1334, Durban, South Africa

**Keywords:** Reality shock, Phenomenology, Newly-graduated radiographers

## Abstract

**Background:**

Globally, the phenomenon of reality shock is a major contributor to the attrition of healthcare professionals. Reality shock negatively impacts on initial workplace transition, productivity, and ultimately, employee retention, hence it is important to ascertain its causative factors so that measures can be taken to mitigate its effects. Relative to other health professions, the field of radiography has been slow in detailing the occurrence of reality shock, and attrition is a major problem affecting the profession. In South Africa, a dearth of data exists pertaining to the potential presence of reality shock amongst newly-graduated radiographers as they transition to the workplace.

**Methods:**

A phenomenological approach was used. Seven newly-graduated radiographers provided their perceptions of their initial workplace experiences. In-depth, one-on-one, face to face interviews were conducted, audio recorded, and transcribed verbatim before interpretive phenomenological analysis was conducted on the obtained data.

**Findings:**

Three main themes emerged relating to increased responsibility, being undermined, and feeling overwhelmed. Respondents felt pressurized by their increased responsibilities when they commenced employment. They also felt undermined by their more experienced colleagues, and they were overwhelmed by the new work routine, which resulted in reality shock.

**Conclusions:**

Curricula at institutions of higher education need to include courses which educate student radiographers on what to expect within the workplace as autonomous practitioners. Heads of imaging departments must create structured induction programs for new employees for adequate orientation and mentoring to reduce reality shock.

**Electronic supplementary material:**

The online version of this article (10.1186/s40359-019-0317-9) contains supplementary material, which is available to authorized users.

## Background

The radiography profession has been experiencing a gradual increase in the number of vacancies globally over the years, a trend which has been replicated in South Africa [1]. Owing to manpower shortages, existing radiographers are overworked as they strive to handle workloads that ideally should have been distributed amongst a much larger workforce [1]. Attrition from the radiography profession is a major cause of this manpower shortage, prompting literature to respond by investigating factors influencing radiographer retention [1]. However, existing literature in this context predominantly focuses on experienced radiographers and factors influencing their retention. This study assumes a different perspective, recognizing that radiographer retention may be heavily influenced by events occurring during their introduction to the professional work environment as autonomous practitioners. During this phase, they may experience reality shock, and this phenomenon has been shown to increase attrition rates amongst health care practitoners [2]. Reality shock is defined as the reactions of newly-qualified workers when they find themselves in work situations which they thought they were prepared for, but suddenly find they are not prepared [3]. Reality shock negatively affects performance, undermines effectiveness, and may result in isolation, overdependence, denial, fear, job dissatisfaction, lack of motivation, and a plethora of other negative emotions and events [4]. It is associated with a greater desire to resign, and in extreme cases, attrition from the profession [2].

The phrase ‘reality shock’ was first reported by Krammer in 1974, after studying the phenomenon in newly-qualified nurses [3]. About three decades later, in 2009, Duchscher concluded a series of qualitative studies performed over a ten-year period, on nurses transitioning into professional practice. Building on Krammer’s study, Duchscher developed the Transition Shock theory which described the stages of reality shock and behaviours exhibited by novice nurses at different points in time during their first year of transition to professional practice [5].

Reality shock has been primarily investigated within the nursing field, hence this profession has well-developed guidelines to prevent this phenomenon, utilising recommendations from studies performed on newly-graduated nurses [6]. Most studies discussing reality shock utilised a variety of qualitative approaches, exploring the experiences of newly-graduated nurses as they transitioned into professional practice to discover challenges preventing a smooth transition [7–9]. They found that nurses experienced reality shock because of the following reasons: they were academically inept; the increased professional accountability of their new roles; pressure to be competent; personal attitudes; and negative interpersonal relationships within the workplace [9]. Intervention strategies to reduce or prevent reality shock directed at universities and hiring hospitals have been suggested [10, 11].

There is scarcity of information detailing the phenomenon of reality shock in newly-qualified radiographers, and this study’s authors were able to identify only a single peer-reviewed article addressing the subject. The article explored expectations and experiences of newly-qualified diagnostic radiographers. The study subjects were all employed at the same imaging department where they had undertaken a year of clinical rotation as students [12]. This study did not find evidence of reality shock, most likely because of the subjects’ prior exposure to their work environment. The three themes that emerged were lack of experience of working out of normal hours; struggling to fit in established groups; and a lack of professional identity and confidence. The study’s authors recommended that further research be undertaken investigating the experiences of new radiography graduates employed at imaging departments where they had no prior work experience as students [12]

In South Africa, no published texts were identified which looked at reality shock in radiographers – a gap this paper aims to fill. Factors contributing to reality shock for local radiographers can only be addressed once they are known and documented, hence the importance of this research which sought to uncover if newly-graduated radiographers experienced reality shock, and if so, what are the contributing factors. This study’s objectives therefore focused on exploring and describing the experiences and expectations of newly-qualified radiographers as they transitioned to professional practice. This was done so that strategies to curb reality shock and improve workplace transition amongst newly-graduated radiographers could be formulated using this information.

## Methods

This qualitative study sought to interview participants in order to understand the meaning they ascribed to their experiences as they transitioned to the work place. Hermeneutic phenomenology was utilised, as the intent was to capture these experiences from the first-person perspective, with the underlying assumption that the context of these experiences was central in understanding the phenomenon.

Ethical clearances were obtained prior to data collection. Participants were newly-graduated radiographers within their first year of employment in Durban, South Africa. Exclusion criteria were prior work experience, and employment for less than three months. Criterion sampling was used to determine five hospitals within the Durban (eThekwini) municipal jurisdiction from which participants would be chosen, based on the South African Department of Health classification system of hospitals (district, regional, tertiary, central, and specialised) [13]. The differences in location of the study participants was necessary for environmental triangulation. A total sampling technique was then used to select all the radiographers at identified hospitals who satisfied the inclusion criteria. Eight radiographers were identified as eligible for study inclusion. A pilot study was conducted by interviewing a respondent who was working at a hospital which had not been selected for inclusion in the study, and no amendments to the interview guide were deemed necessary as all questions were clearly understood and elicited the required responses. The results of the pilot study were not included in the main findings.

A total of seven respondents located at five hospitals consented to study inclusion and were interviewed, and one radiographer declined to participate. Each interview was conducted at a time and place selected by the respondent, in an environment free from disturbance, to encourage uninterrupted flow of conversation. One-on-one, face-to-face, semi-structured, in-depth interviews were conducted by the researcher, and interviews were audio recorded and transcribed verbatim. The interview schedule had seven open-ended questions, and probing questions were posed depending on the responses given by the respondents.

Interpretive phenomenological analysis was conducted on the transcribed data. This was done manually by studying each transcription repeatedly, making notes regarding significant statements (horizontalization), and transforming them into emergent themes which were clustered and labelled. The analysis was systematic and organised, so that information from the data set could easily be located, and traced back to the context of the data. Each step of the analysis was audited and archived for later checking to ensure that the emerging results were as objective as possible. The study’s authors independently analysed the interview transcripts, so that there was investigator triangulation. Data saturation was achieved after analysis of the fifth interview transcript, but the remaining two transcripts were analysed to confirm saturation.

## Results

The demographic data of the interview participants is as shown in Table 1 below.

**Table 1:** Demographic data of participants who were included in this study

Participant number	Sex	Age Range (Years)
1	Female	18–22
2	Female	23–27
3	Female	18–22
4	Female	18–22
5	Male	23–27
6	Female	18–22
7	Female	18–22

Four main themes emerged relating to the reality shock experienced by participants. These are explained below, with quotes from the interviews included to highlight the theme. Names of participants and any other identifying information have been altered to maintain privacy of the respondents and uphold ethical obligations.

(a) Increased responsibility.

Participants highlighted how as autonomous professionals, they felt stressed due to an increased sense of responsibility relative to that which they had experienced as students. They perceived the responsibility of being the sole overseer of the patient’s needs as stressful, because as students they always had assistance, and therefore there was a shared sense of responsibility. It made them nervous to be responsible for certifying that the quality of their own radiographic images was acceptable without consulting someone more experienced, as was the norm during their student phase. They were worried about facing potential liability should they make any mistake. Despite an awareness of these duties prior to commencing autonomous practice, the psychological experience of their new responsibilities brought about reality shock. Participants had the following to say:

“*… it can be a bit stressful, just because as students you don’t really have that much responsibility, but now* [as an autonomous practitioner] *it’s your patient.”* Kelly.

*“I was a bit nervous, because now … there’s no qualified radiographer to ask to pass your image, it’s … it’s just that responsibility where now you’re seen as a qualified.”* Michelle.

*“As students we didn’t take the blame for ourselves…someone had to account for you … now you are here, should you do anything wrong it’s you. You are the one to go under the bus. You cannot point at your tutor … or your fellow student”* Siya.

(b) Being Undermined

Each of the newly-qualified radiographers in this study were working at institutions where there was an existing team of qualified radiographers. As inexperienced members of staff, participants were automatically the most junior staff in the imaging department. Participants experienced reality shock because of the difference between the expectation of how they thought they would be regarded by their qualified peers now that they were no longer students, versus the reality they experienced. Most participants felt taken advantage of, and perceived that their professional role was merely to substitute staff members who were absent due to sickness or other causes. Others felt they were not valued, and subtly undermined by their more experienced peers in the workplace. These sentiments are expressed in the following excerpts:

“*… I think because the older staff … they take advantage of the younger ones because we just came in … And if ever like someone calls in sick … automatically, the younger staff will do* [their duties]*”* Zonke.

“*… sometimes … you feel that…that I’m being undermined a little bit. Because they know you are new”* Michelle.

(c) Feeling overwhelmed.

Participants had expectations of what it would be like to work as autonomous professionals. Their expectations were based on the prior clinical exposure they had obtained as students. However, as students, they were only required to work for a limited time, and they worked under the supervision of a qualified radiographer. As autonomous radiographers who now had to work full-time without supervision, participants were faced with discrepancies between their expectations and the reality they were immersed in, which led to feelings of being overwhelmed. Participants cited different aspects that led to feelings of being overwhelmed, namely the increased workload due to being short-staffed; adjusting to institutional differences between the private and public sector; the routine of coming to work daily; and dealing with the shortages which are found in the public healthcare sector. The following statements highlight these sentiments:

“[We were] *very overwhelmed* [by the workload] *especially because we were very short-staffed. We were like, ‘Woah! What are we in for? Is it gonna be like this? Like how it’s supposed to be?’”* Tina.

*“I had to make like a huge adjustment from the private sector to the government sector.”* Siya.

“*… it was a bit hard … just getting used to the routine of coming to work …*” Kelly.

“*Particularly in public hospitals like this one, where we’re not prepared for the lack of equipment, the lack of money to fix anything, the lack of staffing … there’s just a lack of everything actually.”* Lira.

(d) When participants were asked about their future career prospects, most of them indicated that they were considering leaving radiography. Others said they would like to study further in another qualification to enable them to leave the profession. They had the following to say regarding their career plans:

“*… I am currently maybe thinking of moving out of the field …*” Michelle.

“*… I need to study something else”* Lira.

A sample interview transcript has been supplied (Additional file 1).

## Discussion of themes

When participants were asked to relate their initial workplace experiences, their responses were suggestive of reality shock. This is a multi-faceted phenomenon, and participants expressed differing aspects of their new roles for which they did not feel prepared. They highlighted how the increased responsibilities of being an autonomous professional made them anxious; how frustrated they were due to being undermined; and how the workload overwhelmed them. Such negative sentiments made some participants feel that attrition from the profession was the best way forward.

Participants were aware that once they started working as qualified radiographers, they would assume more responsibility. However, the experience of being immersed in this responsibility brought about reality shock. They expressed the idea of increased responsibilities in a negative way, focusing more on the repercussions of what could happen if they made an error that affected a patient, as opposed to embracing their professional independence.

Study respondents highlighted accepting their own radiographic images as the most significant indicator of their increased level of responsibility. Harvey-Lloyd, Stew and Morris [14] state that the level of responsibility given to practitioners at the outset of their careers is a concern that is acknowledged across the different healthcare professions. Phenomenological research from as early as 1950 describes the anxiety radiographers felt due to the sudden responsibility of accepting their own radiographs [14]. This anxiety may indeed be justified, as radiographer error may have very serious implications for the patient. In one instance in Grimsby in the United Kingdom, a radiographer committed suicide after a barium enema examination he was performing proved fatal for the patient. He had incorrectly inserted a catheter, which perforated the patient’s bowel. Barium from the procedure leaked into the blood stream, and the patient died shortly after the procedure from pulmonary barium micro-embolisation [15].

Naylor [16] documented how repeated studies unanimously found that newly-qualified nurses were unprepared for their increased responsibilities. However, in one study, although surveyed nurses were anxious about their newly acquired responsibility, they felt it gave them ownership of their practice, which is a positive psychological coping mechanism that may be useful to healthcare practitioners in any discipline [16].

Participants in this study felt taken advantage of, because they were viewed as having less personal responsibility due to their young age, as well as being unmarried. As autonomous practitioners, participants expected to be regarded as equal members of staff by their colleagues, but the reality they encountered was that they were undermined in various aspects, which resulted in reality shock. Their colleagues were older, and many were married, or had family responsibilities. These responsibilities would sometimes require them to be absent from work, and the newly-qualified radiographers would be required to take over the duties of the absent staff members. This made participants feel undervalued, and they perceived that they were regarded as substitute staff.

In addition to this, the allocation of duties and rotations were unfavourable to the participants. They were assigned to work during hours that no one else wanted, and to perform duties that the older, qualified staff members preferred not to do. Participants perceived that they were at the very bottom tier in the departmental hierarchy, and they felt they could not protest such treatment as it was never expressly communicated, but rather, subtly implied. Within the nursing profession, such behaviour by qualified staff is described as oppressive, and it is known as horizontal, or lateral violence [16]. This is defined as destructive behaviours of co-workers against one another [17]. Embree and White [18] explain horizontal violence as peer-to-peer aggression, which includes non-verbal innuendo, and undermining activities. Such behaviours are found in what are now termed toxic workplaces. Horizontal violence discourages staff retention, and so the affected newly-graduated radiographers are likely to seek employment elsewhere due to such experiences [17].

Respondents detailed feeling overwhelmed by high workloads upon exposure to their work environments. During clinical rotations as students, there was always supervision, and assistance in dealing with the workload. Now, as unassisted practitioners, they were responsible for ensuring that all patients within the Imaging Department were attended to. They now had to work regularly, for longer hours, and attend to more patients. Additionally, they were short-staffed, further increasing the work pressure. In a separate study, newly-qualified occupational therapists reported being overwhelmed by their schedules, with limited time to complete their professional duties [19]. Naylor echoes this, noting that high patient volumes, heavy workloads, and staff shortages have been cited as the most prevalent sources of work pressure amongst diagnostic radiographers [16]. Continued feelings of being overwhelmed in the workplace can lead to depersonalisation, and this is associated with feelings of detachment, and dehumanisation [20]. Taking time to relax in relaxation rooms and shorter working hours may help radiographers alleviate the overwhelming feelings associated with increased workloads [21].

In this study, most respondents performed their student clinical rotations in the private healthcare sector, but commenced professional practice in public hospitals. In South Africa, there exists a large discrepancy between these two types of institution. Public hospitals generally have longer patient waiting times; shortages of consumables; and compromised patient care due to a high demand for services, and limited healthcare staff. Conversely, private hospitals generally offer better quality patient care, shorter waiting times, and better quality equipment [22]. Participants exposed to the private healthcare system as students reported experiencing reality shock when they were exposed to public hospitals as qualified radiographers. Some of them had almost no exposure to the analogue equipment used in most public hospitals, only being familiar with modern digital equipment. They were unprepared for the shortages staff and consumables, and the bureaucratic, unsupportive management style. Public hospitals within South Africa are reported to be generally in a dysfunctional state, due to bureaucratic bottlenecks [23]. Being immersed in this environment resulted in reality shock for participants.

Respondents in this study experienced reality shock from varying factors, and some of them expressed a desire to leave the radiography profession. Attrition from the radiography profession remains a serious concern in South Africa. The number of radiographers registered by the Health Professions Council of South Africa has been steadily declining over the past few years, underscoring that the attrition rate within radiography in South Africa is alarmingly high [24]. When new healthcare practitioners are exposed to pressure, and adverse events in the working environment, this negatively influences their attitude, satisfaction, and ultimately, increases the likelihood of attrition from the workplace and professional workforce [25]. Research suggests that about 30% of new nurses either change jobs, or leave the profession within their first year of employment due to reality shock [10]. In this study, although some participants were still deliberating on leaving radiography, one participant had already taken action to this end, and was awaiting admission into university to study a programme in a different field.

However, the findings of this research are not without pitfalls. The results of this study may have limited transferability, as is true for all qualitative studies. Another limitation is that the interviews were conducted by a radiographer, and thus interpretations may be biased towards the interviewer’s own experiences within the radiography field, as opposed to reflecting the participants’ true sentiments.

## Recommendations

Managing reality shock involves multiple stakeholders, and this process ideally should begin with institutions of higher learning. The recommendations outlined in this study are an amalgamation of solutions to challenges faced by respondents, as well as insights provided by literature and the authors’ knowledge of the subject.

Universities should structure clinical rotations for students such that every individual is exposed to a low-resource, public hospital as part of the standard requirements. This will enable students to familiarise themselves with such clinical settings. The curriculum should also include a module for final year students which explains to them how the professional experience will differ from their student experience, utilising data from studies such as this one.

In addition, students should be encouraged to apply for their first job at hospitals where they have worked during their clinical rotation as students. When this is not possible, the student must familiarise themselves with their prospective places of work by informal visits, asking peers for information, talking to existing staff members at the hospital, and so on. The familiarity with the surroundings and staff will assist in reducing reality shock.

Management and senior staff members at hospitals recruiting newly-qualified graduates should be formally trained on mentoring, orientation, and encouraging retention of new members of staff. It is important that the rest of the existing staff is also taught how to relate to new staff members, so that a welcoming environment is created.

New radiographers must be given a reduced workload, which is gradually increased as their competency levels rise. Initially, they should work for less hours, and attend to few patients so that they work at a decreased pace. Over time, this ought to then be reviewed depending on individual competency, and the patient load and working hours increased accordingly. Such ‘easing in’ of new employees should help in reducing reality shock, and thereby reduce the likelihood of attrition from the workplace and workforce by newly-qualified radiographers.

## Conclusion and further research

Early career experiences tend to remain entrenched in an individual’s mind for several years, and may subsequently influence important decisions, such as choosing to leave a profession. This paper has identified and suggested relatively simple measures which may help to prepare newly-qualified radiographers for the workplace, which should in turn decrease the impact of reality shock. However, recruiting a larger sample of respondents from a different location may yield additional valuable insights which this study may have failed to harness. It is therefore a recommendation for further research that a similar study be conducted with more respondents, and from different provinces in the country. In addition, data collection should ideally be longitudinal, so that the changing needs of respondents at different points in time can be observed, documented, and possibly catered to.

Institutions of higher learning and health care facilities should consider implementing these recommendations as a step in combating the problem of radiographer attrition due to reality shock.

## Additional file

Additional file 1: ANNEXURE 1. Sample interview transcript. (DOCX 18 kb)
