# Supplementary material for: Reality shock in radiography: fact or fiction? Findings from a phenomenological study in Durban, South Africa
Source: BMC Psychol. 2019 Jun 25;7:40. doi: 10.1186/s40359-019-0317-9 (PMC6593581; doi:10.1186/s40359-019-0317-9)
Supplement: Supplementary file 1 — ANNEXURE 1. Sample interview transcript. (DOCX 18 kb) [file 40359_2019_317_MOESM1_ESM.docx]

ANNEXURE 1: Sample interview transcript

Participant Number: XX

Interview Date: 21/08/2018

**Section A:** Demographic Data

**Data not shown due to ethical reasons.**

**Section B:** Interview

**Interviewer**: Greeted participant and introduced self as well as explaining the purpose of the interview.

**Interviewer**: May I begin with the interview?

**Participant**: ***Okay.***

**Main research question:**

**Interviewer**: How would you describe your experiences as a qualified radiographer in their first year of professional practice?

**Participant: *Experiences? Erm… ok there’s experiences?* [Laugh] *It’s just…very...as your first year out of university it’s very different to how they prepare you, let me put it that way… it’s a lot more in-depth, because you’re here every day, whereas as a student, you’re not here every day. As a student you are here some nights, some days, but now you’re hands-on, and yeah… it’s a lot more intense I would say, because now you… you actually have to take everything you’ve learnt to practice it every single day, different situations that you weren’t exposed to as a student.***

**Interviewer**: And personally, would you say it’s been an enjoyable experience, are you on the fence, or has it been more of a negative experience?

**Participant**: ***Errm, I’m on the fence* [laughs]. *I would say, you enjoy some aspects, but some aspects you don’t. Particularly in public hospitals like this one, where we’re not prepared for the lack of equipment, the lack of money to fix anything, the lack of staffing is just a general-there’s just a lack of everything actually. So your experience it sometimes is enjoyable ’cause you do nice work, but at other times it’s very difficult when you don’t have things to work with.***

**Interviewer**: Right, and how is the lack of like equipment and staffing, how does it impact your overall experience here?

***Participant: It definitely leaves a negative taste on the government healthcare system, let’s put it that way. That the government healthcare system is definitely failing the population, to put it that way. Erm, as opposed to… we used to work in private as students, so you can see why things work in private, and why things don’t work in government.***

**Interviewer**: Alright, and as a comm serve, what do you feel should have been done to make your comm serve experience a better one?

**Participant*: Better experience? Err… I think before applying for hospitals, erm… when you do the application process, you just get a name that you just apply to. You don’t get a full idea of what’s… you’re actually going for. And I do think all the government hospitals should actually have an assessment before they are put up as training facilities… for students, for comm serves, for anything.***

**Interviewer**: So during your, erm… student phase, you said you trained in a private hospital?

**Participant**: ***Erm, mixed so… some… there were eight different ones but… so some private, some government.***

**Interviewer**: Oh… okay. And how about your technical skill? How do you feel your student experience technically relates to your comm serve experience?

**Participant**: ***Erm… as a student, I was lucky in that technically I had to practice more than I can here, because I’m limited here, and we don’t have digital equipment here-you saw the darkroom. We don’t have fluoroscopy, we don’t have… we only have CT and general. Those are the only two things you can practice here, whereas as a student I still got to practice everything.***

**Interviewer**: And in terms of rotations, how are those as a comm serve?

**Participant**: ***Rotations, we don’t rotate much, there’s not much to rotate to* [laugh].**

**Interviewer**: What do you do?

**Participant**: ***There’s literally… we have CT, general, orthopaedics, er… orthopaedics just happens to be digital, but it’s not fully digital, er… but we don’t have screening, we don’t have… oh we have theatre, but not here – at XXXX hospital, but we don’t have as much rotation as we should be having for experience.***

**Interviewer**: Right, and how about stuff like public holidays and nights, do you have to work those as well as a comm serve?

**Participant**: ***Yes. You are bottom of the log* [laughs], *you work everything… you… they wait seven years here for Christmas leave so…you’re not gonna get public holidays… yeah***.

**Interviewer**: Alright… and workload? How would you describe that relative to being a student? I know you did kinda touch on it but maybe just elaborate…

**Participant**: ***Workload is… it actually just depends here… that’s strange because you sit some days and you do nothing, and other days we… depending on the staffing situation, we run around all day. So actually yeah, it’s very dependant.***

**Interviewer**: Alright, so what would you say... What are the key aspects of your job that you’d say you like?

**Participant**: ***I like? Erm… I like the fact that we get a lot of different cases here, it’s actually trauma cases and that kinda thing, we never see the same thing twice, we really do see interesting things here* [laughs].**

***Er…and I do like that we have some… it’s not always a good thing, but particularly as a comm serve here you can make decisions that you probably couldn’t make as a comm serve anywhere else. Er…because of the lack of… sort of… management. So you very much have to be hands-on here, which is quite enjoyable in a way… it’s not necessarily a good thing.***

**Interviewer**: So, are you saying it’s like increased responsibility?

**Participant**: ***Yes, because you have no one to help you here* [laughs].**

**Interviewer**: Alright, and the key things that you dislike?

**Participant**: ***Dislike? On that note, I don’t like that there’s no one to help you when you actually do need it… management-wise it’s very inefficiently run here. If something breaks, or something needs to be moved, or a patient can’t be done, you make that decision as a radiographer, whereas that actually should be a management thing.***

**Interviewer**: And this is you, as a comm serve?

**Participant**: ***As a comm serve, yeah. I do think management… but I think in most of the hospitals that is a problem.***

**Interviewer**: How about your relationships with other… with the broader hospital staff. How do you find those, between you as a comm serve radiographer and all others, like doctors, nurses, everyone else?

**Participant**: ***Ermm I find certain departments – orthopaedics you deal with the doctors quite a lot, and theatre obviously with the doctors, and trauma. But besides that, we don’t relate to the rest of the hospital at all. I don’t see anyone else, I don’t know who anyone else is…* [laughs].**

**Interviewer**: So you probably… if you met them on the street you probably wouldn’t have any…

**Participant**: ***No, I would have no idea, no. Unless I’d seen them… maybe trauma we see the same doctors, but…***

**Interviewer**: And how about in terms of support, how would you rate the level of support that you’ve received as a comm serve?

**Participant**: ***From co-workers? A lot…the staff that have been here you know, ten, twenty years. They’ve really…in the beginning of the year they do…they try and explain things and help you out. In terms of management and things – nothing. We get no support, erm… we don’t even do orientation at the beginning of the year…. we don’t do any of that.***

**Interviewer**: So you just jump right into it?

**Participant**: ***Yeah, on your first day it’s like no we’ll do orientation later, and they never do it.***

**Interviewer**: Alright, and how about five years from now. Where do you see yourself?

**Participant**: ***Five years from now I need to study something else. [laugh] Sounds terrible.***

**Interviewer**: Within radiography-within the health field….?

**Participant**: ***No, within radiography, but I don’t know about diagnostic radiography…. I don’t...yeah. Within, but definitely something…I actually don’t know yet…but something different.***

**Interviewer**: Is there anything else that you’d like to add that you think…maybe that we didn’t touch on that you think might help me get a clearer picture of your experiences at…as a comm serv.

**Participant**: ***Ermmm… I’m trying to think… experiences as a comm serve erm… it’s very… it’s very difficult… yeah yeah… I’m not too sure what you’re… what kinda information you’re looking for…***

**Interviewer**: No, no… just general… like I said, no right or wrong answers… it’s an individual thing.

**Participant**: ***I do think overall a comm serve experience is important, if I can put it that way. However, in this particular institution being a comm serve doesn’t mean you’re a comm serve, it means you’re a qualified ten years, that you have to behave like you’ve been qualified for ten years. You… you have to… whereas I think… other people, a comm serve experience is more… you should be a junior still. But just in this particular case we very much kinda have to… sort yourself out.***

**Interviewer**: Thanked the participant once more for their time, and ended the interview session.
